# Comparative Efficacy of Neoadjuvant Endocrine Therapy, Neoadjuvant Chemotherapy, and Neoadjuvant Chemo-Endocrine Therapy in Estrogen Receptor–Positive Breast Cancer Patients: A Meta-Analysis

**DOI:** 10.1155/tbj/1670410

**Published:** 2025-05-15

**Authors:** Yi Yuan, Ning Cui, Ziyi Xu, Chang Cui, Zongpeng Zhou, Zhefu Ma

**Affiliations:** Breast Plastic Surgery, Liaoning Cancer Hospital & Institute of China Medical University, Shenyang, China

**Keywords:** breast cancer, clinical response, estrogen receptor–positive, neoadjuvant chemotherapy, neoadjuvant endocrine therapy, pathological complete response

## Abstract

Neoadjuvant therapy before surgery offers varying benefits as a well-established treatment option for breast cancer. This study specifically evaluated the effectiveness of neoadjuvant endocrine therapy (NET), neoadjuvant chemotherapy (NCT), and neoadjuvant chemo-endocrine therapy (NCET) in patients with estrogen receptor (ER)–positive breast cancer. This meta-analysis was conducted and reported in accordance with the Preferred Reporting Items for Systematic Reviews and Meta-Analyses (PRISMA) guidelines. Electronic searching was conducted to retrieve articles from databases including PubMed, Cochrane Library, EMBASE, CNKI, and Wanfang. The primary outcome measured by odds ratios (ORs) with 95% confidence intervals (CIs) focused on assessing pooled effect sizes. Random-effects or fixed-effect models were conducted according to the existence of statistical heterogeneity. A total of 15 eligible articles were included in the analysis. The results indicated clinical response (CR) (OR = 0.54; 95% CI = 0.41 to 0.73; *I*^2^ = 39.6%) and clinical complete response (cCR) (OR = 0.31; 95% CI = 0.12 to 0.85; *I*^2^ = 68.0%) after NET was significantly higher than NCT. However, no significant difference was shown in pathological complete response (pCR) (OR = 0.49; 95% CI = 0.23 to 1.04; *I*^2^ = 0.0%) and breast-conserving surgery (BCS) (OR = 0.49; 95% CI = 0.23 to 1.04; *I*^2^ = 0.0%). The combined paradigm of NCET presented no significant improvement compared with monotherapy of NET or NCT. Overall, both NET and NCT are effective neoadjuvant treatment options for patients with ER+ breast cancer. More explicit clinical decision indicators need to be further clarified. And NCET does not offer additional benefits over NET or NCT in patients with ER+ breast cancer.

## 1. Introduction

Breast cancer is now recognized as the most commonly diagnosed cancer globally and ranks as the fifth leading cause of cancer-related deaths. Recent estimates reported approximately 2.3 million new cases and 685,000 fatalities annually [1]. The cornerstone modalities in the multifaceted treatment of breast cancer, including surgery, chemotherapy, radiotherapy, endocrine therapy, and targeted therapy, have significantly contributed to improved survival rates. Notably, approximately 75% of breast cancer patients are identified as hormone receptor–positive [2], often referred to as the luminal tumor where endocrine therapy plays an essential role in treating this type. With the advancement in treatment, there has been a growing emphasis on individualizing therapy in clinical management. Consequently, treatment decisions guided by tumor subtypes and lymph node involvement have the potential to optimize treatment effectiveness, avoiding both overtreatment and undertreatment.

Neoadjuvant therapy has gained prominence as a pivotal presurgical treatment for estrogen receptor (ER)–positive patients, presenting a similar clinical prognosis compared with adjuvant therapy [3, 4]. Neoadjuvant therapy includes neoadjuvant endocrine therapy (NET), neoadjuvant chemotherapy (NCT), and neoadjuvant chemo-endocrine therapy (NCET). One of the key benefits of neoadjuvant therapy is its ability to reduce tumor size, thereby increasing the likelihood of successful breast-conserving surgery (BCS) [5, 6]. Recognized as a standard of care, neoadjuvant therapy has demonstrated substantial clinical efficacy. For instance, previous studies have reported that NET yielded a similar clinical response rate (cRR) to NCT while exhibiting lower toxicity [7]. According to the current ASCO guidelines, neoadjuvant systemic therapy should be offered to patients with high-risk HER2-positive or triple-negative breast cancer (TNBC) patients [8]. Based on the CSCO guidelines, NET may be considered in patients with hormone receptor–positive (HR+) breast cancer patients who require preoperative neoadjuvant therapy but are not candidates for chemotherapy, are temporarily ineligible for surgery, or do not require immediate surgery. NET may also be considered for those hormone-dependent patients who are insensitive or less effective to NCT [9].

The integration of chemotherapy with NCET remains a subject of debate [10]. While certain studies have indicated that NCET did not lead to a significant improvement in the pathological complete response (pCR) rate [11], others found that NCET showed a higher clinical complete response (cCR) and pCR rate than chemotherapy alone in postmenopausal women with acceptable toxicity [12]. Despite various meta-analyses evaluating the effectiveness of NET and NCT in breast cancer, such as those by Wang et al., which highlight the superior tumor response with NCT in hormone receptor-positive cases, and by Huang et al., who support the continued recommendation of NCT despite NET's benefits, few studies compared the effectiveness of NCET and NET/NCT. Therefore, the aim of our study is to conduct a meta-analysis comparing the efficacy of NET, NCT, and NCET in ER+ breast cancer patients.

## 2. Materials and Methods

This study was conducted and reported in strict accordance with the Preferred Reporting Items for Systematic Reviews and Meta-Analyses (PRISMA) guidelines [13]. We registered the protocol for this meta-analysis on the PROSPERO website, with the registration identification number CRD42022351611.

### 2.1. Search Strategy

Comprehensive database searches were performed including PubMed, EMBASE, Cochrane Library, CNKI, and Wanfang databases (from the inception to June 16, 2022). The search keywords include breast cancer, breast neoplasm, breast tumor, breast carcinoma, locally advanced breast cancer, NET (treatment), neoadjuvant therapy, NCT, and neoadjuvant chemo-endocrine therapy (treatment). No restrictions were placed on language. Manual searches of references from retrieved articles ensured the inclusion of all relevant studies.

### 2.2. Selection Criteria

The inclusion criteria were as follows: (1) adult patients diagnosed with ER+ breast cancer (Population); (2) studies that evaluated the effectiveness of NET, NCT, or NCET (Intervention and Comparator); (3) studies reporting at least one of the following outcomes (Outcomes): cRR, cCR rate, pCR rate, and BCS rate; and (4) prospective or retrospective design studies (Study design). Exclusions were made for case reports, abstracts, reviews, letters, and comments. Screening and assessment of the identified articles were carried out by two independent researchers. Differences in opinions between the two reviewers were reconciled through discussion, and when required, a third reviewer was consulted for resolution.

### 2.3. Data Extraction

Two independent authors extracted data from each included study in our meta-analysis. Relevant data covering first author name, publication year, a detailed regimen of treatment, sample size in each treatment group, ER status, HER-2 status (±), menopausal status (pre/postmenopausal), and clinical outcomes were systematically extracted using a standardized template. Clinical primary outcomes included cRR, cCR rate, pCR rate, and BCS rate. Consensus with all investigators was used to resolve all discrepancies in our study.

### 2.4. Outcomes

The outcomes included cRR, cCR, pCR, and BCS rates. According to the Response Evaluation Criteria in Solid Tumors (RECIST) criteria, cRR has either cCR or clinical partial response (cPR). The cCR was defined as the complete disappearance of all assessable breast lesions during the physical examination. The pCR was defined as either the complete disappearance of all invasive tumors in the breast and lymph nodes or the absence of residual invasive cancer upon hematoxylin and eosin evaluation of the completely resected breast specimen and all sampled regional lymph nodes (ypT0/Tis ypN0) [14].

### 2.5. Study Quality Assessment

The Jadad score was utilized for assessing the methodological quality of the studies included [15]. The scale focuses on randomization (0–2 points), blinding (0–2 points), and withdrawal/dropouts (0-1 point). Total scores ranged from 0 to 5, with a score of 3 or greater considered to be of high quality. The Cochrane Risk Assessment Tool ROB2.0 was used for bias assessment [16]. Studies were categorized as high, low, and unclear risk based on the results of a comprehensive assessment of five domains.

### 2.6. Statistical Analysis

Statistical analyses were performed using Stata V.14.0 (Stata Corp LP) software.

The odds ratio (OR) with 95% confidence intervals (CIs) was used for estimating pooling effect sizes. Cochrane Q statistics and an *I*^2^ test were used to calculate the heterogeneity between eligible articles of our study. *I*^2^ > 50% indicates the existence of heterogeneity. Random-effects or fixed-effect models were used according to the statistical heterogeneity. If substantial heterogeneity among the studies was observed, a random-effects model was applied as our primary analytical approach. Subgroup analysis was used to explore the sources of heterogeneity. Furthermore, publication bias was systematically assessed using funnel plots and Egger's test. All statistical analyses were two-sided, with statistical significance established at a *p* value threshold of less than 0.05.

## 3. Results

### 3.1. Search Results

**Figure 1:**
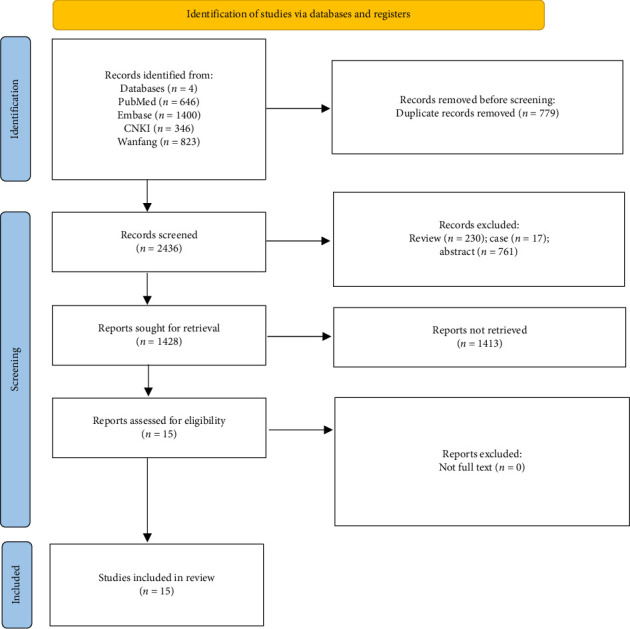
Flowchart for search results and selection details.

The selection process for eligible studies is presented in Figure 1. A total of 3215 articles were identified, from which 779 duplicates were removed. Subsequent screening excluded 1008 articles based on study types. After screening the abstracts and titles (removing 1413 articles) and reviewing the full texts, a total of 15 studies were included [11, 12, 17–27].

### 3.2. Study Characteristics

The included studies' characteristics are outlined in Table 1. Our meta-analysis included 1296 ER+ breast cancer patients. There were seven included studies targeted on postmenopausal patients, one on premenopausal patients, and four on both premenopausal and postmenopausal. The publication period ranged between 2012 and 2021. Of these, 11 articles evaluated the efficacy of NET versus NCT, three compared NCT versus NCET, and one compared NET versus NCET. The primary clinical outcomes included cRR, cCR, pCR, and BCS rate.

### 3.3. Quality Assessment

**Figure 2:**
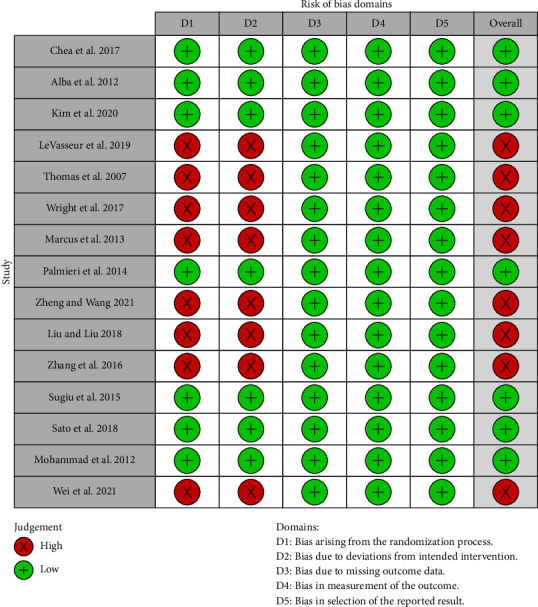
Risk bias assessment of the included studies using the ROB2 tool.

The quality assessment by the ROB2 tool and Jadad score of the included studies is shown in Figure 2 and Supporting Table 1. Eight studies exhibited a high risk of bias related to the randomization process and deviations from the intended intervention. The risk of bias concerning missing outcome data, selection of reported results, and measurement of outcomes was low across all studies. Furthermore, the evaluation results derived from the Jadad scale indicated that none of the studies included in the analysis employed a blinding methodology.

### 3.4. Results of Meta-Analysis

#### 3.4.1. Clinical Response (CR)

**Figure 3:**
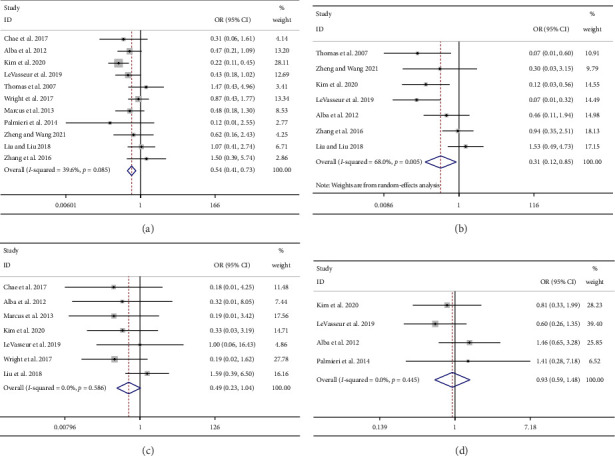
The cRR (a), cCR (b), pCR (c), and BCS (d) rate of neoadjuvant endocrine therapy versus neoadjuvant chemotherapy in breast cancer patients. cRR, clinical response rate; cCR, clinical complete response; pCR, pathological complete response; BCS, breast-conserving surgery.

Eleven studies reported the numbers of ER+ breast cancer patients who achieve CR after NET or NCT treatment. Significantly higher numbers of CR patients were observed in the NCT group than in the NET group (OR = 0.54; 95% CI = 0.41 to 0.73; *I*^2^ = 39.6%; Figure 3(a)).

**Figure 4:**
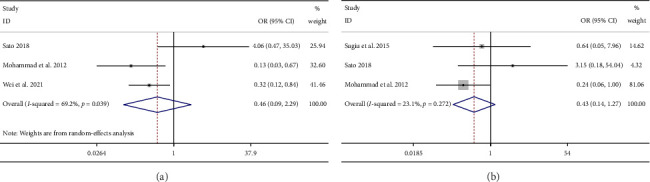
The cRR (a) and pCR (b) rate of monotherapy (neoadjuvant endocrine therapy or neoadjuvant chemotherapy) versus neoadjuvant chemo-endocrine therapy in breast cancer patients. cRR, clinical response rate; cCR, clinical complete response; pCR, pathological complete response.

Furthermore, three studies (NET vs. NECT: *n* = 1 [27]; NCT vs. NECT: *n* = 2 [10, 28]) presented the numbers of CR patients after NET/NCT treatment alone or NCET combination therapy. Based on high heterogeneity, our findings suggested that for CR numbers, there was no significant difference between those two therapies (OR = 0.46; 95% CI = 0.09 to 2.29; *I*^2^ = 69.2%; Figure 4(a)).

#### 3.4.2. cCR

Seven studies reported the numbers of cCR patients between NET and NCT in ER+ breast cancer treatment. The meta-analysis results showed that the numbers of cCR patients were significantly higher in the NCT treatment group than in the NET group (OR = 0.31; 95% CI = 0.12 to 0.85; *I*^2^ = 68.0%; Figure 3(b)).

#### 3.4.3. pCR

Seven studies reported the number of patients with pCR after NET or NCT treatment. No significant difference in pCR numbers was observed between NET and NCT treatment (OR = 0.49; 95% CI = 0.23 to 1.04; *I*^2^ = 0.0%; Figure 3(c)). Moreover, three studies (NET vs. NECT: *n* = 1 [27]; NCT vs. NECT: *n* = 2 [9, 10]) assessed the number of patients achieved pCR between NET/NCT and NCET, and our findings suggested no significant difference in pCR numbers between these two treatments (OR = 0.43; 95% CI = 0.14 to 1.27; I^2^ = 23.1%; Figure 4(b)).

#### 3.4.4. BCS

Only four studies reported the number of patients who received BCS after NET and NCT. Our results showed the BCS difference between NET and NCT was not significant (OR = 0.93; 95% CI = 0.59 to 1.48; *I*^2^ = 0.0%; Figure 3(d)).

### 3.5. Results of Subgroup Analysis

The results of the subgroup analysis are presented in Table 2. Regarding CR, the pooled results for HER-2− subgroups were consistent with the original analysis, with fewer CR patents in the NET group than in the NCT group (OR = 0.50; 95% CI = 0.36 to 0.69; *I*^2^ = 41.1%). The results of HER-2± subgroup analysis showed that there was no significant difference in the number of CR patients between the NET group and the NCT group (OR = 0.83; 95% CI = 0.40 to 1.71; *I*^2^ = 32.9%). For menopausal status, the postmenopausal subgroup analysis showed no significant difference in CR between the NET group and the NCT group (OR = 0.68; 95% CI = 0.44 to 1.06; *I*^2^ = 0.0%). In addition, there was only one published study for each of the premenopausal and pre/postmenopausal subgroups, which does not support meaningful meta-analysis at present.

Contrary to the original analysis results, both HER-2± and HER-2− subgroup analyses showed no difference in the number of cCR patients between the NCT group and the NET group (HER-2±: OR = 0.31; 95% CI = 0.02 to 3.96; *I*^2^ = 79.3%; HER-2−: OR = 0.30; 95% CI = 0.09 to 1.02; *I*^2^ = 69.5%). However, HER-2 status subgrouping did not reduce the heterogeneity of cCR analysis. For menopausal status, the results of NR subgroup analysis were consistent with the original analysis (OR = 0.14; 95% CI = 0.04 to 0.54; *I*^2^ = 47.2%), and the results of postmenopausal subgroup analysis were contrary to the original analysis (OR = 0.95; 95% CI = 0.22 to 4.08; *I*^2^ = 33.7%), with reduced levels of heterogeneity in both subgroups.

As for pCR, the results of the HER-2− subgroup analysis were consistent with the results of the original analysis, that is, there was no difference in the number of pCR patients between the NET and NCT groups (OR = 0.52; 95% CI = 0.24 to 1.16; *I*^2^ = 0.0%). Subgroup analysis based on menopausal status showed that the results of postmenopausal subgroup (OR = 0.50; 95% CI = 0.21 to 1.21; *I*^2^ = 28.6%) and NR subgroup (OR = 0.59; 95% CI = 0.08 to 4.53; *I*^2^ = 0.0%) were consistent with the results of the original analysis, and there was only one study in the premenopausal subgroup.

Regarding the number of BCS patients, the results of HER-2 status subgroup analysis (HR-2-: OR = 0.90; 95% CI = 0.56 to 1.45; *I*^2^ = 16.9%) and menopausal status subgroup analysis (NR: OR = 0.94; 95% CI = 0.53 to 1.66; *I*^2^ = 57.1%) were consistent with the results of the original meta-analysis, and the heterogeneity between the results was increased only in the NR subgroup.

Because the number of studies comparing the difference in efficacy between NCET and NET/NCT is small, the available data only supported the subgroup analysis of CR and pCR. The heterogeneity decreased when grouping according to HER-2 status. And the results of HER-2± subgroup analysis showed that the number of CR patients in the NECT group was higher than that in the NCT/NET group (OR = 0.25; 95% CI = 0.11 to 0.58; *I*^2^ = 0.0%), and the results of pCR were consistent with the original analysis (OR = 0.30; 95% CI = 0.09 to 1.03; *I*^2^ = 0.0%). Based on menopausal status, NR subgroup analysis showed that there was no difference in the number of CR between the NCET group and the NCT/NET group (OR = 0.94; 95% CI = 0.08 to 11.08; *I*^2^ = 78.5%). One study in the postmenopausal subgroup suggested that the number of CR in the NCET group was less than that in the NCT/NET group (OR = 0.13; 95% CI = 0.03–0.67).

### 3.6. Publication Bias and Sensitivity Analysis

Among NET and NCT, potential publication bias was observed in pCR analysis according to the funnel plot (Figures S1–S4). However, Egger's and Begg's test indicated no publication bias in CR, cCR, pCR, and BCS analysis (*p* > 0.05). The results of sensitivity analysis confirmed the robustness of the results of BCS and cCR analysis. Sensitivity analysis identified one study that could be a source of potential heterogeneity among CR [19, 29] and pCR [23, 30] related studies, respectively (Figures S5–S8).

Furthermore, potential publication bias was shown in CR analysis by funnel plot (Figures S9 and S10). Although the existence of publication bias was not confirmed by Egger's and Begg's tests, the results of sensitivity analysis suggested that the CR and pCR analysis of NCET versus NET/NCT was not robust (Figures S11 and S12).

## 4. Discussion

While NCT and NET are increasingly employed in operable breast cancer and have demonstrated substantial clinical efficacy [28, 32], researchers have further explored the subdivision indications and combined treatment options of these two treatment methods. Published studies have shown that patients with ER+ breast cancer have different responses to NCT and NET due to differences in study publication time, different drugs, dosing regimens, and patient characteristics [15, 17, 20, 21, 24, 33]. Also, in the exploration of NCET, more significant cell proliferation reduction was observed when rhythmic cyclophosphamide (i.e., frequent, low-dose cyclophosphamide) was combined with letrozole than letrozole alone [34]. However, studies that compared the efficacy of NCET and NET/NCT in ER+ breast cancer patients were still limited, and only one known systematic review analyzed the efficacy of three different neoadjuvant regimens in terms of pCR and overall response rate (ORR) in HR-positive breast cancer. The results of our meta-analysis and the analysis of Bottini (2006) both suggested that the CR indicators (such as CR and cCR) were better for NCT than for NET in both HR+ and ER+ breast cancer patients, but the difference between combination therapy and monotherapy was not significant. The main difference between these two meta-analyses is that Bottini (2006) considered that the pCR rate of NET patients was significantly lower than that of NCT patients, while both our meta-analysis and subgroup analysis results showed that the pCR difference between the NET group and the NCT group was not significant. At the same time, considering the importance of the popularization of neoadjuvant therapy in the treatment of breast cancer to increase the proportion of BCS, we innovatively found that there were no differences for NET and NCT in improving the number of patients who could undergo BCS treatment.

Avoiding axillary lymph node dissection is an important advantage and purpose of neoadjuvant therapy for patients with tumor-positive lymph nodes turning negative. Studies have shown that 20%–40% of breast cancer patients can achieve axillary lymph node downstaging after NCT treatment, and in HER2-positive patients, this proportion can even be higher than 50% [29, 35, 36]. Two prospective trials, ACOSOG Z1071 and SENTINA, which used dual tracers to collect three or more sentinel lymph nodes, confirmed the fact that the false-negative rate after NCT treatment was very low [37, 38]. The results of the included studies and the combined analysis also showed that a higher CR in the NCT group was clearly more advantageous in solving the problem of lymph node dissection.

Although the results of our meta-analysis showed that NCT led to a better CR, pCR and CR measures were not effective surrogate endpoints for tumor survival. In addition to axillary lymph node dissection, for patients with ER+ breast cancer, downstaging of the tumor by neoadjuvant systemic therapy and allowing patients who would otherwise require mastectomy [39] to receive BCS are also important considerations. From the results of the meta-analysis, there was no difference in the BCS rate between the NET and NCT groups. This may illustrate the need for clearer clinical indications for the selection of NCT and NET in patients who need to undergo radical mastectomy for breast cancer.

As a variety of endocrine therapy drugs, such as tamoxifen, fulvestrant, and aromatase inhibitors (AIs), have shown significant efficacy in patients with ER+ breast cancer, the advantages of low toxicity and high tolerance of endocrine therapy have received extensive attention. A promising CR of NET in breast cancer women with postmenopausal or low baseline Ki-67 was confirmed by multiple studies [24, 33, 40, 41]. However, only a few studies which targeted some carefully selected premenopausal patients can be observed with positive NET efficacy [42, 43]. Similar to the results of our meta-analysis, the small sample GEICAM study analyzed the response rate of chemotherapy versus exemestane in premenopausal breast cancer patients, and the results showed that the chemotherapy group was higher than the endocrine therapy group [18]. The most significant problem with NET is the long time it takes to achieve the maximum response. The study by Lombart-Cussac et al. showed that the proportion of patients who achieved a maximum response after 6 months was 37% [33]. Another study observed that the median time to BCS of patients receiving NET intervention was 7.5 months [44]. Therefore, the NCT intervention group may preferentially reach the maximum response for the same observation time with NET.

Based on the results of the current data analysis, the NECT combination regimen may be a high-risk and low-benefit option for ER+ breast tumors. Our results showed that neither CR nor pCR was significantly superior with NECT combination therapy compared with NET or NCT single-class drug therapy. Meanwhile, the results of HER-2± subgroup analysis even showed that the number of CR patients in the NECT group was lower than that in the NET/NCT group. More importantly, the safety of the combination has been questioned by some clinical trials, and the results of previous studies have shown that the safety of NECT, especially in the incidence of high-grade (≥ Grade 3) adverse events, is significantly higher than that of NET alone [26, 45]. For postmenopausal women with breast cancer, this is difficult to accept.

### 4.1. Limitations

Although this study provides a reliable and comprehensive analysis, it has some limitations. First, many eligible studies included are relatively small in sample sizes, which might limit the informative value of comparisons. Second, the number of studies reporting CR and pCR numbers was limited between NCET and NET/NCT groups. Despite this limitation, these studies were included to provide a more comprehensive overview. Finally, a publication bias was identified in CR and pCR analysis. Therefore, our results were interpreted with caution.

## 5. Conclusions

In the present study, ER-positive breast cancer patients after NCT may have better CR and cCR than those after NET, and no significant difference was observed in BCS and pCR. Further research is imperative to elucidate the surrogate relationship between response rates and long-term survival outcomes, necessitating more comprehensive survival data.

## Figures and Tables

**Table 1:** Characteristics of the eligible studies.

Study	Sample size			Intervention	Control	ER status	HER-2 status	Menopausal status	Outcomes
	NET	NCT	NCET						
Chae et al. 2017 [17]	13	12	—	NET	NCT	+	HER-2±	Postmenopausal	SUVmax; pCR; cRR

Alba et al. 2012 [18]	48	47	—	NET	NCT	+	HER-2−	NR	BCS, cCR, cRR, and pCR measured by MRI

Kim et al. 2020 [19]	87	87	—	NET	NCT	+	HER-2−	Premenopausal	BCS, cCR, cRR, and pCR assessed by MRI

LeVasseur et al. 2019 [20]	51	111	—	NET	NCT	+	HER-2−	NR	BCS, cCR, cRR, and pCR
						+			

Thomas et al. 2007 [21]	53	38	—	Letrozole	NCT	+	HER-2±	NR	cCR, cRR, and pCR based on biopsy
						+			

Wright et al. 2017 [22]	57	83	—	NET	NCT	+	HER-2−	Postmenopausal	cRR and pCR based on biopsy
						+			
						+			
						+			

Marcus et al. 2013 [23]	27	72	—	NET	NCT	+	HER-2−	Postmenopausal	cRR and pCR based on biopsy

Palmieri et al. 2014 [24]	22	22	—	Letrozole	FE100C	+	HER-2±	Postmenopausal	BCS and cRR based on ultrasound or mammogram
						+			

Zheng and Wang 2021 [25]	20	20	—	Letrozole or tamoxifen	Epirubicin + cyclophosphamide followed by docetaxel	+	HER-2−	Pre/postmenopausal	cCR and cRR based on ultrasound

Liu and Liu 2018 [28]	70	46	—	Letrozole	Paclitaxel chemotherapy	+	HER-2−	Postmenopausal	cRR and pCR based on imaging and pathology
						+			

Zhang et al.2016 [29]	42	44	—	Letrozole	FEC: epirubicin + cyclophosphamide fluorouracil; EC: Epirubicin + cyclophosphamide; TC: docetaxel + cyclophosphamide	+	HER-2±	Pre/postmenopausal	cCR and cRR based on biopsy
						+			

Sugiu et al. 2015 [11]	—	12	16	NCT combination with LHRHagonist or AI	NCT	+	HER-2±	Pre/postmenopausal	pCR based on biopsy

Sato 2018 [26]	14	—	42	Exemestane	Exemestane + cyclophosphamide	+	HER-2−	Postmenopausal	pCR and cRR based on biopsy

Mohammad et al. 2012 [11]	—	30	32	NCT + letrozole	5-Fluorouracil, doxorubicin, and cyclophosphamide	+	HER-2±	Postmenopausal	pCR and cRR based on biopsy and histopathologic examination
						+			
						+			

Wei et al. 2021 [27]	—	39	39	Docetaxel + cyclophosphamide	Docetaxel + cyclophosphamide + letrozole	+	HER-2±	Pre/postmenopausal	cRR and cCR

Abbreviations: MRI, magnetic resonance imaging; NCET, neoadjuvant chemo-endocrine therapy; NCT, neoadjuvant chemotherapy; NET, neoadjuvant endocrine therapy; NR, not reported.

**Table 2:** Results of the subgroup analysis.

Subgroup	*N*	Pooled estimates (95% CI)	Heterogeneity	
			*I* ^2^ (%)	*p* value
NET versus NCT				
Clinical response (CR)				
HER-2 status				
HER-2±	4	0.83 (0.40, 1.71)	32.9	0.215
HER-2−	7	0.50 (0.36, 0.69)	41.4	0.115
Menopausal status				
Postmenopausal	6	0.68 (0.44, 1.06)	0.0	0.563
Premenopausal	1	0.22 (0.11, 0.45)	—	—
NR	3	0.57 (0.33, 0.97)	31.9	0.23
Pre/postmenopausal	1	1.50 (0.39, 5.74)	—	—
Clinical complete response (cCR)				
HER-2 status				
HER-2±	2	0.31 (0.02, 3.96)	79.3	0.028
HER-2−	5	0.30 (0.09, 1.02)	69.5	0.011
Menopausal status				
Postmenopausal	2	0.95 (0.22, 4.08)	33.7	0.219
Premenopausal	1	0.12 (0.03, 0.56)	—	—
NR	3	0.14 (0.04, 0.54)	47.2	0.151
Pre/postmenopausal	1	0.94 (0.35, 2.51)	—	—
Pathological complete response (pCR)				
HER-2 status				
HER-2±	1	0.18 (0.01, 4.25)	—	—
HER-2−	6	0.52 (0.24, 1.16)	0.0	0.523
Menopausal status				
Postmenopausal	4	0.50 (0.21, 1.21)	28.6	0.241
Premenopausal	1	0.33 (0.03, 3.19)	—	—
NR	2	0.59 (0.08, 4.53)	0.0	0.600
Breast-conserving surgery (BCS)				
HER-2 status				
HER-2±	1	1.41 (0.28, 7.18)	—	—
HER-2−	3	0.90 (0.56, 1.45)	16.9	0.3
Menopausal status				
Postmenopausal	1	1.41 (0.28, 7.18)	—	—
Premenopausal	1	0.81 (0.33, 1.99)	—	—
NR	2	0.94 (0.53, 1.66)	57.1	0.127
NCET versus NCT/NET				
Clinical response (CR)				
HER-2 status				
HER-2±	2	0.25 (0.11, 0.58)	0.0	0.370
HER-2−	1	4.06 (0.47, 35.03)	—	—
Menopausal status				
Postmenopausal	1	0.13 (0.03, 0.67)	—	—
NR	2	0.94 (0.08, 11.80)	78.5	0.031
Pathological complete response (pCR)				
HER-2 status				
HER-2±	2	0.30 (0.09, 1.03)	0.0	0.517
HER-2−	1	3.15 (0.18, 54.04)	—	—

## Data Availability

The data presented in this study are available in the article or supporting information.

## Supporting Information

Supporting Information 1 Figure S1: The funnel plot in the cRR of in ER+ breast cancer. (NET vs. NCT). cRR, clinical response rate.

Supporting Information 2 Figure S2: The funnel plot of the cCR rate in ER+ breast cancer. (NET vs. NCT). cCR, clinical complete response.

Supporting Information 3 Figure S3. The funnel plot of the pCR rate in ER+ breast cancer. (NET vs. NCT). pCR, pathological complete response.

Supporting Information 4 Figure S4: The funnel plot of the BCS rate in ER+ breast cancer. (NET vs. NCT). BCS, breast-conserving surgery.

Supporting Information 5 Figure S5: The funnel plot of the cRR rate in ER+ breast cancer. (NET vs. NCET). cRR, clinical response rate.

Supporting Information 6 Figure S6: The funnel plot of the cCR rate in ER+ breast cancer. (NET vs. NCET). cCR, clinical complete response.

Supporting Information 7 Figure S7: The funnel plot of the pCR rate in ER+ breast cancer. (NET vs. NCET). pCR, pathological complete response.

Supporting Information 8 Figure S8: Sensitivity analysis of the BCS rate in ER+ breast cancer. (NET vs. NCT). BCS, breast-conserving surgery.

Supporting Information 9 Figure S9: The funnel plot of the CR rate in ER+ breast cancer. (NCET vs. NCT/NET). CR, clinical response.

Supporting Information 10 Figure S10: The funnel plot of the pCR rate in ER+ breast cancer. (NCET vs. NCT/NET). pCR, pathological complete response.

Supporting Information 11 Figure S11: Sensitivity analysis of the CR rate in ER+ breast cancer. (NCET vs. NCT/NET). CR, clinical response.

Supporting Information 12 Figure S12: Sensitivity analysis of the pCR rate in ER+ breast cancer. (NET vs. NCT). pCR, pathological complete response.

Supporting Information 13 Table S1. Study quality based on the Jadad score.
