# Supplementary figures and images for: Comparative Efficacy of Neoadjuvant Endocrine Therapy, Neoadjuvant Chemotherapy, and Neoadjuvant Chemo-Endocrine Therapy in Estrogen Receptor–Positive Breast Cancer Patients: A Meta-Analysis
Source: Breast J. 2025 May 15;2025:1670410. doi: 10.1155/tbj/1670410 (PMC12097862; doi:10.1155/tbj/1670410)

Funnel plot with pseudo 95% confidence limits

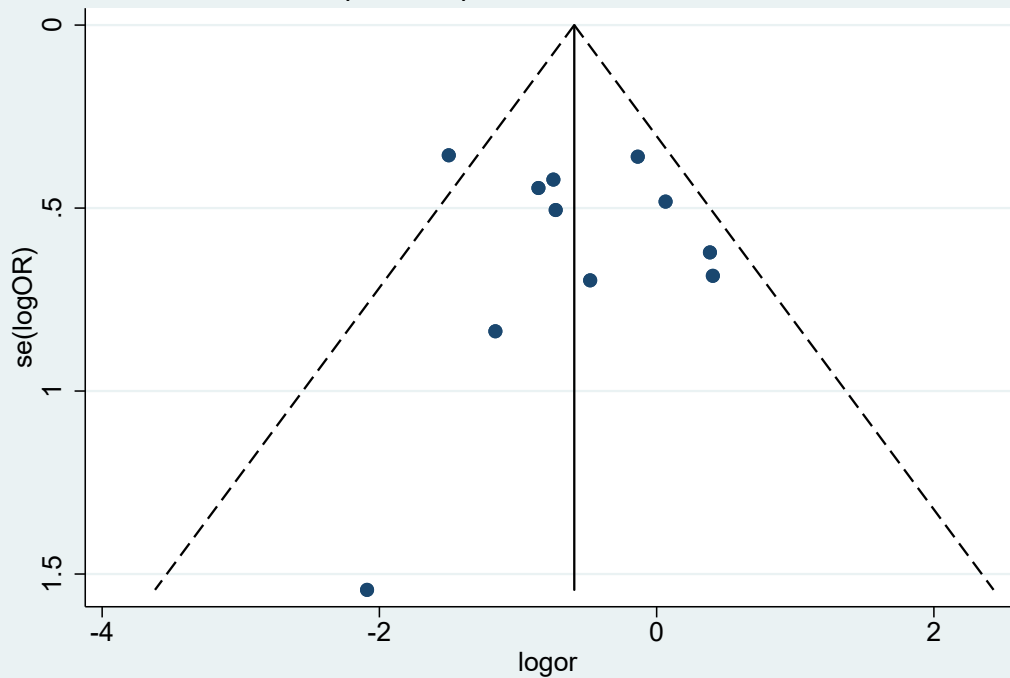

Supplement: Supporting Information 1 — Figure S1: The funnel plot in the cRR of in ER+ breast cancer. (NET vs. NCT). cRR, clinical response rate. [file 1670410.f1.pdf]

Funnel plot with pseudo 95% confidence limits

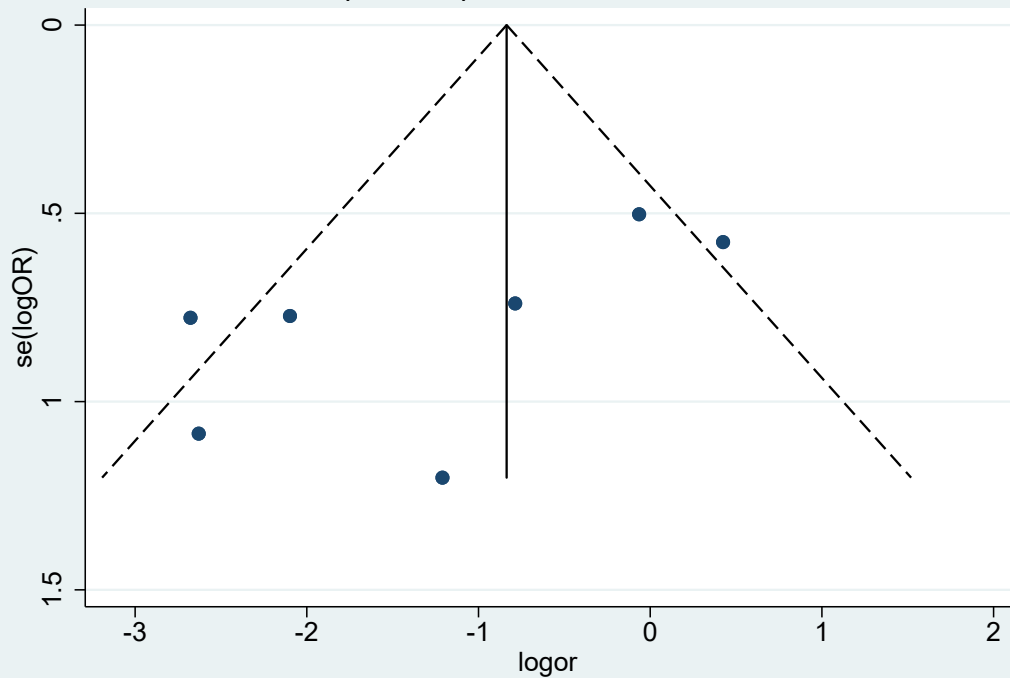

Supplement: Supporting Information 2 — Figure S2: The funnel plot of the cCR rate in ER+ breast cancer. (NET vs. NCT). cCR, clinical complete response. [file 1670410.f2.pdf]

Funnel plot with pseudo 95% confidence limits

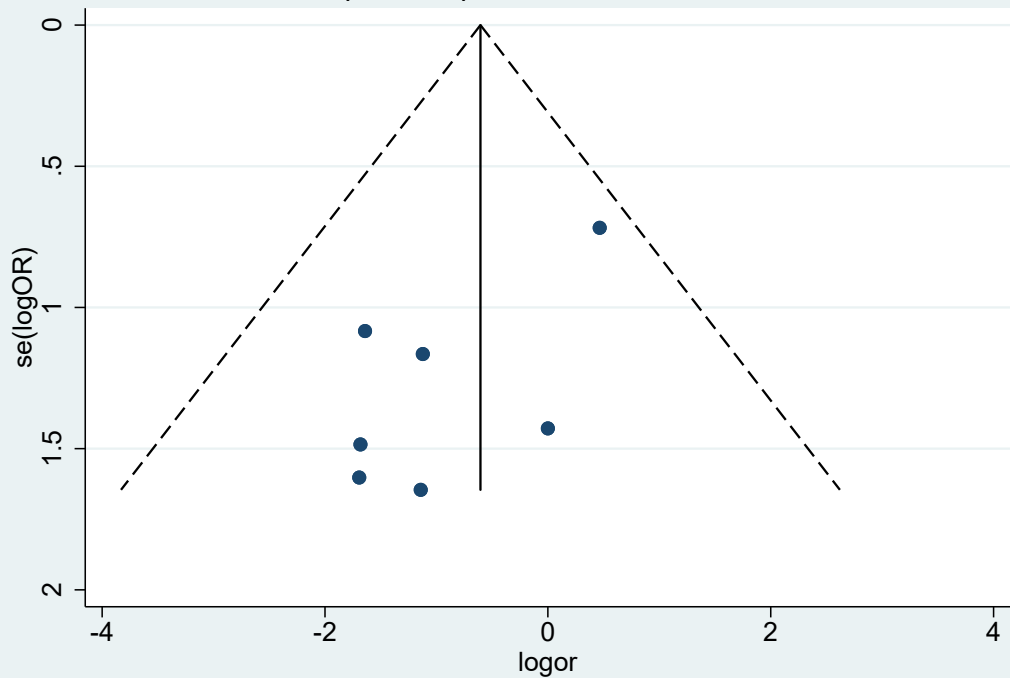

Supplement: Supporting Information 3 — Figure S3. The funnel plot of the pCR rate in ER+ breast cancer. (NET vs. NCT). pCR, pathological complete response. [file 1670410.f3.pdf]

Funnel plot with pseudo 95% confidence limits

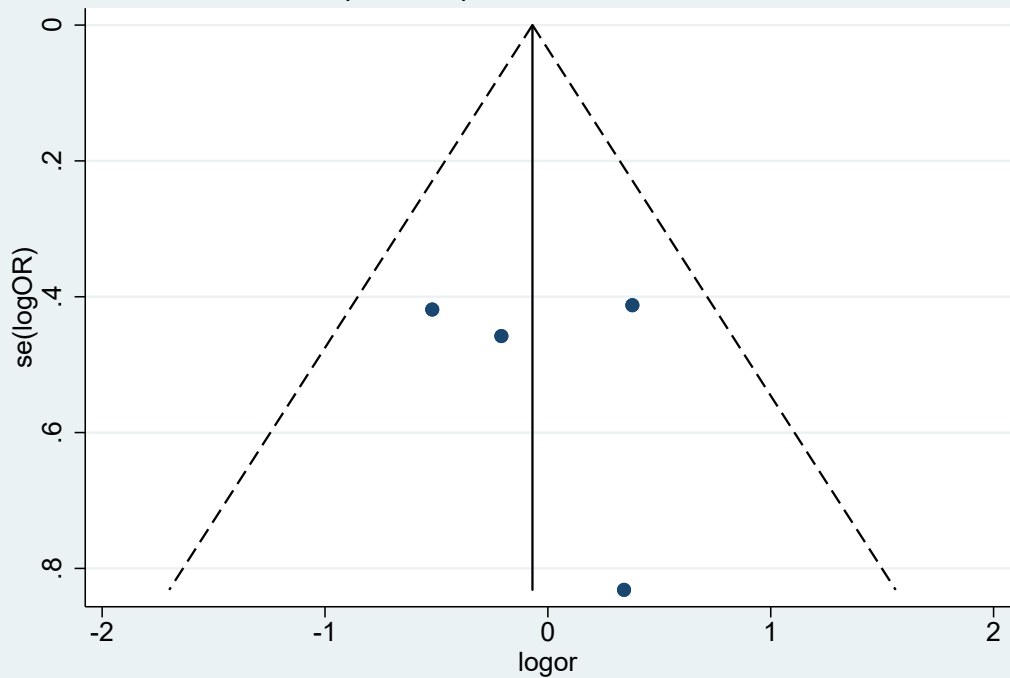

Supplement: Supporting Information 4 — Figure S4: The funnel plot of the BCS rate in ER+ breast cancer. (NET vs. NCT). BCS, breast-conserving surgery. [file 1670410.f4.pdf]

Funnel plot with pseudo 95% confidence limits

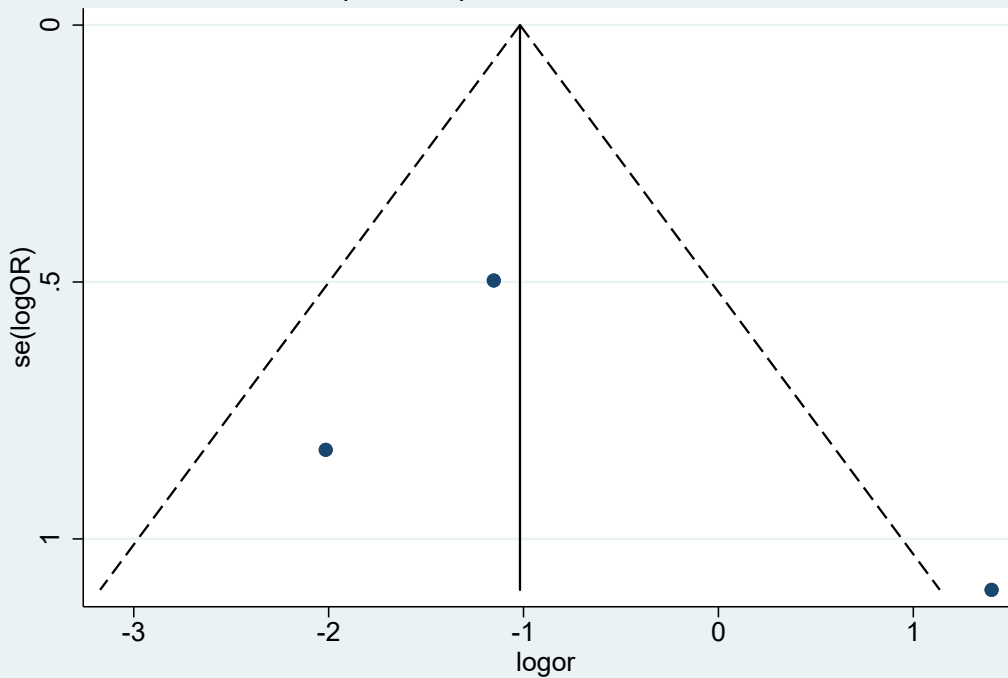

Supplement: Supporting Information 9 — Figure S9: The funnel plot of the CR rate in ER+ breast cancer. (NCET vs. NCT/NET). CR, clinical response. [file 1670410.f9.pdf]

Funnel plot with pseudo 95% confidence limits

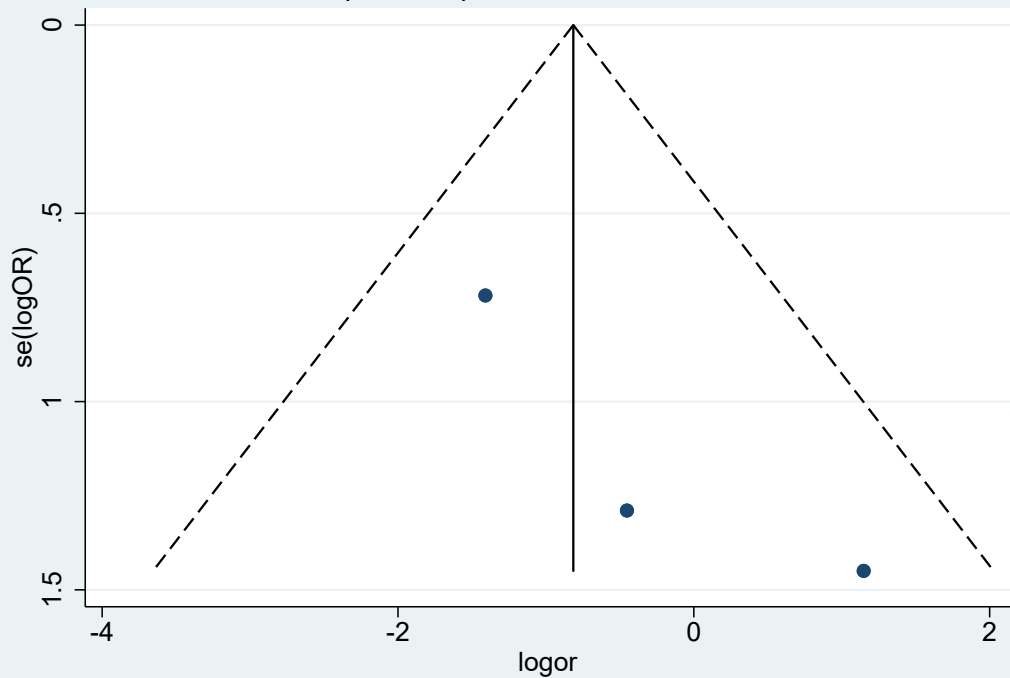

Supplement: Supporting Information 10 — Figure S10: The funnel plot of the pCR rate in ER+ breast cancer. (NCET vs. NCT/NET). pCR, pathological complete response. [file 1670410.f10.pdf]
