# Supplementary material for: Comparative Efficacy of Neoadjuvant Endocrine Therapy, Neoadjuvant Chemotherapy, and Neoadjuvant Chemo-Endocrine Therapy in Estrogen Receptor–Positive Breast Cancer Patients: A Meta-Analysis
Source: Breast J. 2025 May 15;2025:1670410. doi: 10.1155/tbj/1670410 (PMC12097862; doi:10.1155/tbj/1670410)
Supplement: Supporting Information 13 — Table S1. Study quality based on the Jadad score. [file 1670410.f13.docx]

**Table S1.** Study quality based on Jadad score.

| Study | Randomization | Blind | Withdrawal/Dropouts | Totle score |
| --- | --- | --- | --- | --- |
| Chea et al 2016 | 2 | 0 | 1 | 3 |
| Alba et al 2012 | 2 | 0 | 1 | 3 |
| Kim et al 2020 | 2 | 0 | 1 | 3 |
| LeVasseur et al 2019 | 1 | 0 | 1 | 2 |
| Thomas et al 2007 | 1 | 0 | 1 | 2 |
| Wright et al 2015 | 1 | 0 | 1 | 2 |
| Marcus et al 2013 | 1 | 0 | 1 | 2 |
| Palmieri et al 2014 | 1 | 0 | 1 | 2 |
| Zheng et al 2021 | 2 | 0 | 1 | 3 |
| Liu et al 2018 | 1 | 0 | 1 | 2 |
| Zhang et al 2016 | 1 | 0 | 1 | 2 |
| Sugiu et al 2015 | 2 | 0 | 1 | 3 |
| Sato et al 2018 | 2 | 0 | 1 | 3 |
| Mohammad et al 2012 | 2 | 0 | 1 | 3 |
| Wei et al 2021 | 1 | 0 | 1 | 2 |

**Randomization:** Lack of explicit reference to "randomness" limited description thereof (0 point); Mentions "random" but fails to provide a description of the randomization method employed (1 point); A suitable randomization method was clearly articulated (2 point).

**Blind:** No mention of blinding or a detailed description of the blinding process was inappropriate (0 point); The term "double-blind" was referenced, but its implementation was not explained (1 point); Provide a clear description of how the blind method is implemented (2 point).

**Withdrawal/Dropouts:** No information about withdrawals or dropouts was provided (0 point); A clear report of the number and reasons for withdrawals and dropouts (1 point).
